# Beyond clinical risk: tackling loneliness through a population health lens

**DOI:** 10.3389/fpsyg.2025.1609060

**Published:** 2025-08-18

**Authors:** Ananda Zeas-Sigüenza, Pablo Ruisoto, Kami Koldewyn, Ferran Muntané, Joan Benach

**Affiliations:** ^1^Department of Health Sciences, Public University of Navarre, Pamplona, Spain; ^2^Developmental Social Vision Lab, Department of Psychology, School of Psychology and Sport Science, Bangor University, Bangor, Wales, United Kingdom; ^3^I-COMMUNITAS, Institute for Advanced Social Research, Public University of Navarre, Pamplona, Spain; ^4^IdisNA, Navarre Institute for Health Research, Public University of Navarre, Pamplona, Spain; ^5^JHU-UPF Public Policy Center (JHU-UPF PPC), UPF Barcelona School of Management (UPF-BSM), Universitat Pompeu Fabra (UPF), Barcelona, Spain; ^6^Research Group on Health Inequalities, Environment, and Employment Conditions (GREDS-EMCONET), Department of Political and Social Sciences, Universitat Pompeu Fabra, Barcelona, Spain

**Keywords:** loneliness, social isolation, social connection, social processing, clinical risk, population intervention, public health

## Abstract

Loneliness is a distressing emotional state that arises from unmet social needs, particularly the *quality*—rather than merely the *quantity*—of social connections. While it serves an adaptive function by signaling social disconnection and motivating reconnection, chronic loneliness is a well-established risk factor for adverse mental and physical health outcomes. Recognizing its growing prevalence and health burden, the World Health Organization (WHO) has identified loneliness as a public health priority. To date, most research and interventions have focused on high-risk individuals, mainly within clinical populations, often addressing loneliness only after it becomes severe and entrenched. This paper argues for a paradigm shift toward population-level interventions that targets the broader social and environmental determinants of loneliness. Specifically, we propose a loneliness spectrum model and a systemic intervention framework that targets structural determinants, positioning loneliness prevention as a fundamental public health strategy through nature-based and community-driven interventions.

## 1 Introduction

Loneliness is a distressing emotional state that arises when an individual’s social needs—particularly the need for meaningful connection—are unmet (Cacioppo and Patrick, 2008). When this signaling mechanism fails to resolve disconnection, loneliness can become chronic and harmful (Cacioppo and Cacioppo, 2018). However, when left unaddressed, loneliness can become chronic, triggering a self-perpetuating cycle of social withdrawal, psychological distress, and eventually, physical health problems (Cacioppo and Patrick, 2008). Even prior to the COVID-19 pandemic, loneliness had emerged as a significant societal concern, with 1.8 million people in the UK and one in three individuals across European Union reporting feelings of loneliness (Arsenijevic and Groot, 2018; ONS, 2021). The COVID-19 pandemic exacerbated these trends, further underscoring the urgency of addressing loneliness as a public health priority (World Health Organization, 2020). This paper introduces a spectrum-based conceptualization of loneliness and a systemic intervention framework to inform policy and practice. Here, the loneliness spectrum refers to a continuum spanning from transient, adaptive social disconnection to chronic, maladaptive loneliness characterized by neurobiological desensitization and persistent health risks.

## 2 Loneliness and health outcomes

Loneliness activates the neurobiological stress response, signaling the need for social reconnection (Cacioppo et al., 2014). However, when individuals are unable to regulate this response, prolonged loneliness can trigger its chronic dysregulation, with detrimental effects on health. For example, a comprehensive meta-analysis involving over 20,000 individuals found a significant association between loneliness and systemic inflammation, including elevated levels of interleukin-6 (IL-6), a pro-inflammatory cytokine, even after controlling for confounding variables (Smith et al., 2020). Similarly, research by Hawkley and Cacioppo (2003) highlights how chronic loneliness can disrupt the hypothalamic–pituitary–adrenal (HPA) axis, leading to excessive cortisol production, immune suppression, and increased inflammatory markers such as C-reactive protein (CRP) and IL-6. These findings underscore the role of loneliness in exacerbating susceptibility to infection and chronic disease through sustained immune and endocrine dysregulation.

Indeed, the long-term health consequences of loneliness can be profound for both individuals and society. Social isolation consistently results in increased likelihood of mortality, whether measured objectively or subjectively. Holt-Lunstad et al. (2015) conducted a meta-analysis of 70 studies including over 3 million participants followed for an average of 7 years. They found that mortality risk increased by 26% for loneliness, 29% for social isolation, and 32% for living alone—even after adjusting for depression, socioeconomic status, health behaviors, and demographics. The averaged effect sizes remained of similar magnitude irrespective of the covariates that were or were not included in the models (*p* > 0.20), including covariates relevant to depression, socioeconomic status, health status, physical activity, smoking, gender, and age (Holt-Lunstad et al., 2015). In addition to premature death, loneliness is strongly associated with higher risk of morbidity, contributing to cardiovascular disease (Valtorta et al., 2016), sleep fragmentation (Kurina et al., 2011), and a 31% increased likelihood of developing dementia (Luchetti et al., 2024). Unsurprisingly, loneliness is also a strong predictor of depression (Cacioppo et al., 2006), and shares a bidirectional relationship with perceived stress, whereby each amplifies the other (Laustsen et al., 2024). Both depression and chronic stress, in turn, have their own negative impacts on physical health, compounding the burden of disease. When combined, these effects place a considerable strain on health and care systems (National Academies of Sciences, Engineering, and Medicine, 2020). Yet, two critical gaps remain in the evidence base. First, there is a need to clarify how the severity (the intensity of symptoms) and chronicity (requiring longitudinal data analysis of symptoms experienced and/or the number of episodes) of loneliness affect health outcomes (Käll et al., 2020c). Second, a more comprehensive account of the biopsychosocial mechanisms linking loneliness impacts brain function, bodily health, and social behavior is needed. For example, urban environments with limited public spaces and high residential turnover can erode social networks, while socioeconomic inequality restricts opportunities for meaningful connection and reinforces stigma and exclusion.

Despite its far-reaching health implications, the relevance of social connection to public health has received limited attention, likely due to decades of siloed development of evidence across disciplines such as epidemiology, psychology, and sociology (Holt-Lunstad, 2022). Moreover, the role of loneliness and social (dis)connection as social determinants of health inequities remain largely unexplored, calling for a more integrated population health approach (Benach and Muntaner, 2025).

## 3 Loneliness and the processing of social information

A recent study by Tomova et al. (2020) found that craving social interaction activates the same brain regions—specifically substantia nigra and ventral tegmental area—involved in food craving. Interestingly, this neural activation was absent in individuals experiencing severe loneliness, suggesting that it may suppress the brain’s social craving response. This finding supports the idea that loneliness functions as an adaptive mechanism, akin to hunger—a fundamental biological drive that motivates individuals to seek connection, just as hunger drives food intake. Typically, this mechanism motivates reconnection and subsides once social needs are met. However, in cases of severe loneliness, this mechanism may become dysregulated, desensitizing the brain to the rewarding aspects of social interaction. This neurobiological adaptation process can reinforce social withdrawal and create a self-perpetuating cycle of isolation.

Social (re)connection is widely recognized as a protective health factor. A landmark meta-analysis of 148 studies, involving 308,849 participants across North America, Europe, Asia, and Australia, found that individuals with stronger social relationships had a 50% increased likelihood of survival, regardless of age, sex, initial health status, cause of death, or follow-up duration (Holt-Lunstad et al., 2010). These findings suggest that the impact of social relationships on mortality is comparable to well-established risk factors such as smoking and alcohol consumption and even exceeds the influence of physical inactivity and obesity (Holt-Lunstad et al., 2010). However, critical unresolved questions remain: how and why do supportive or rewarding social interactions deteriorate and become aversive? At what specific threshold does loneliness shift from a beneficial self-regulatory mechanism to a harmful, self-perpetuating cycle? How can social connection be prioritized in social policies to reduce health inequities?

Beyond diminishing social motivation, loneliness also alters social information processing. Lonely individuals exhibit a cognitive bias toward perceiving ambiguous social interactions as threatening (Spithoven et al., 2017), which can be further aggravated by social inequality axes, such as social class or gender (Salas Quijada et al., 2023). Furthermore, studies have found that loneliness is associated with reduced activation in the ventral striatum (a region involved in reward processing) and the temporoparietal junction (TPJ; involved in Theory of Mind), and heightened activation in early visual cortex when viewing social stimuli (Cacioppo et al., 2009). According to the Loneliness Loop Model (Cacioppo and Hawkley, 2009) and the Social Information Processing (SIP) Model (Spithoven et al., 2017), loneliness can induce a state of hypervigilance to social threats, increasing individuals’ sensitivity to rejection and reducing the likelihood of engaging in positive social interactions. These alterations of social perception and social motivation can, in turn, perpetuate social disconnection and deepen feelings of isolation.

Further neuroimaging research on loneliness has identified changes in brain regions associated with social cognition, such as the left posterior superior temporal sulcus (pSTS) (Kanai et al., 2012a, 2012b), posterior TPJ, and the prefrontal cortex (Nakagawa et al., 2015). Two systematic reviews (Cacioppo and Cacioppo, 2012; Lam et al., 2021) have identified three core neural networks involved in loneliness: (1) the social perception network, including regions such as the pSTS and TPJ; (2) the reward system, particularly the ventral striatum; and (3) the threat processing system, primarily the amygdala. These neurobiological insights underscore the importance of designing population-level interventions that preempt chronic dysregulation by fostering environments conducive to social connection, thus reducing the burden before individual health problems emerge.

Together, these findings point to a complex interplay between neurobiological, cognitive, and social processes, reinforcing the need for interventions that target not only individual behaviors but also the structural conditions that shape social (dis)connection.

## 4 Interventions aimed to reduce loneliness

Despite growing recognition of loneliness as a public health issue, most research and intervention efforts have focused on high-risk individuals, particularly those experiencing severe or chronic loneliness. While such clinical approaches are necessary, preventive strategies targeting the general population remain underdeveloped, even though they offer substantial potential for reducing the overall burden of loneliness (Rose, 1992; Rose et al., 2008). Framing loneliness as an adaptive mechanism—akin to hunger or stress—provides a crucial framework for designing effective population-level interventions. This perspective acknowledges loneliness as a biopsychosocial experience rather than simply a clinical state. Moreover, emerging evidence that social interactions can have neuroprotective effects reinforce the case for universal, upstream strategies that promote meaningful connection before loneliness becomes chronic. In line with this approach, Holt-Lunstad’s (2022) calls for a paradigm shift: moving beyond person-centered interventions toward context-centered and universal strategies that reshape the environments in which loneliness arises. To advance this shift, a more comprehensive, context-sensitive model is needed, mapping interventions along the loneliness spectrum by considering both severity and chronicity as key factors. Such a model would allow for targeted prevention and support across different levels of risk, from promoting everyday social engagement to addressing persistent, health-threatening isolation.

### 4.1 A spectrum-based approach to loneliness and health

Traditional loneliness research has often relied on a binary classification—lonely versus non-lonely—that fails to capture the fluid, temporal nature of loneliness. In reality, loneliness unfolds along a continuum or spectrum, ranging from brief, situational episodes to persistent, health-threatening states. At the adaptive end of the spectrum, transient loneliness functions as a helpful signal, motivating individuals to reconnect socially. In these cases, the stress response is regulated, and the experience is often short-lived. As loneliness becomes more episodic or recurrent, individuals may begin to show signs of emotional distress and vulnerability, but the response remains potentially reversible with targeted support. At the chronic end, however, prolonged social disconnection may lead to neurobiological desensitization, reduced sensitivity to social rewards, dysregulated stress responses, and increased risk of mental and physical health problems. This progression is not linear, nor universal, but highlights how severity and chronicity interact to shape the impact of loneliness.

Recognizing this dynamic spectrum allows for more nuanced and equitable responses—tailoring interventions not only to individual symptoms but also to structural and social factors that determine where people fall along this continuum.

A major limitation in current research is the tendency to measure loneliness at a single time point, overlooking the role of chronicity—the duration and persistence of loneliness. This distinction is critical: while some individuals may experience acute but short-lived loneliness, others may suffer from chronic loneliness for years. Without incorporating chronicity, interventions may fail to differentiate between transient and persistent loneliness, limiting their effectiveness—especially when applied to population-level strategies. To address these gaps, a more dynamic and multidimensional research approach is needed—one that combines longitudinal data, neurobiological markers, and social-behavioral patterns. Such an approach would allow researchers and policymakers to better identify when and why loneliness becomes maladaptive, and to determine how these transitions may be shaped by social stratification, driving health inequities. This would strengthen efforts to develop responsive, equitable interventions tailored to individuals and communities at varying points along the loneliness spectrum.

To address these gaps, future studies should incorporate longitudinal cohort designs, ecological momentary assessment (EMA), and randomized controlled trials (RCTs) that integrate neurobiological, behavioral, and contextual data to track loneliness trajectories and intervention effects over time.

### 4.2 Expanding interventions beyond the individual: leveraging contextual factors

Current interventions for loneliness have primarily focused on individual-level psychological strategies, such as cognitive-behavioral therapy (CBT) (Käll et al., 2020a, 2020b, 2021; Käll and Andersson, 2023) and mindfulness (Creswell et al., 2012). While these approaches have demonstrated efficacy, a recent meta-analysis (Zeas-Sigüenza et al., 2025) suggests they are most effective for individuals experiencing moderate to severe loneliness. This raises the question of whether broader, context-based interventions could help prevent loneliness from reaching clinical severity.

Emerging research suggests that nature-based and community-driven interventions may be particularly effective in reducing loneliness exposure. For example, access to green and blue spaces has been associated with a 28% reduction in loneliness (Astell-Burt et al., 2022; Hammoud et al., 2021). The effectiveness of such interventions depends not only on proximity to nature but also on the presence of structured opportunities for meaningful social interaction. Recent systematic reviews have identified promising but mixed evidence suggesting that nature-based interventions may help reduce loneliness and enhance social connectedness, although effect sizes and methodological quality vary considerably across studies (Astell-Burt et al., 2022; Bettmann et al., 2025; Leavell et al., 2019). Addressing loneliness effectively requires more than facilitating new contacts—it involves cultivating a sense of belonging and mutual support (Haslam et al., 2022).

This calls for a paradigm shift away from the individual-deficit model—which frames loneliness because of personal maladaptive thoughts or behaviors—toward a context-sensitive model that recognizes loneliness as the product of both individual and social-structural determinants. To achieve this shift, interventions must integrate structural and social determinants of health, aiming to create settings that naturally support social connection among populations and specific social groups.

In this context, the Flourishing and Wellbeing Initiative provides a compelling interdisciplinary framework to be adapted for addressing loneliness through social-structural interventions. By tackling social inequalities in access to resources and opportunities, the initiative advances social justice while engaging multiple stakeholders—including academia, workplaces, public institutions, and community organizations (NIHROHBRvC, n.d.).

This framework advocates for nature-based interventions and community-driven initiatives designed to foster meaningful social connections while promoting collective well-being. *Nature-based interventions* leverage outdoor settings to encourage social engagement, personal growth, and environmental stewardship through physical and environmentally sustainable activities. *Community-driven initiatives*, such as time banks, mutual aid networks, and local participation schemes, are grassroots efforts that empower residents to co-create solutions tailored to their unique cultural and contextual needs. Moreover, by identifying universal intervention principles, this framework lays a foundation for designing sustainable, context-sensitive interventions that not only mitigate loneliness but also promote individual and collective well-being. For instance, nature-based interventions such as community gardening, group nature walks, and the RECETAS program in Europe have shown promising results in reducing loneliness and strengthening social ties, with measurable improvements in loneliness scores and mental health outcomes (Vert et al., 2024). Singapore’s Community Networks for Seniors have also demonstrated reductions in loneliness and improvements in health outcomes through coordinated outreach, home visits, and community activities (Malhotra et al., 2018; Ministry of Health Singapore, 2019). In the United Kingdom, Social Prescribing models integrate primary care with community resources to enhance social connection and well-being (Drinkwater et al., 2019; Polley et al., 2017). Outcomes across these approaches include reductions in standardized loneliness measures such as the UCLA Loneliness Scale (Russell, 1996), improvements in biomarkers of stress and inflammation (e.g., cortisol, interleukin-6) (Cacioppo and Cacioppo, 2014; Smith et al., 2020), and enhanced indicators of social cohesion (Kawachi and Berkman, 2000). Policymakers, healthcare providers, and community organizations each play a vital role in designing, funding, implementing, and evaluating these strategies to ensure they are equitable, culturally sensitive, and responsive to diverse population needs. However, the scalability of these interventions remains contingent upon sustainable funding, cross-sector collaboration, and cultural adaptability. For example, while RECETAS shows promise in European cities, its replication in lower-resource contexts may face infrastructural or institutional challenges.

## 5 Reframing loneliness as a public health issue

Based on the growing prevalence and profound social and health impacts of loneliness, the World Health Organization (WHO) has declared loneliness a public health priority (World Health Organization, 2020). Yet, current research and policy responses have disproportionately focused on high-risk individuals, particularly within clinical populations, often intervening only once loneliness has become severe and entrenched. This narrow focus neglects the broader social determinants that shape loneliness across the general population—and particularly among social groups exposed to structural vulnerabilities (Holt-Lunstad, 2022).

We propose an urgent paradigm shift: from treating loneliness as a personal affliction to addressing it as a collective, socially patterned challenge. This requires implementing population-level interventions that tackle the root causes of loneliness, thereby improving population health and health equity (Benach et al., 2013). Nature-based and community-driven initiatives exemplify this shift, offering promising avenues for mitigating loneliness and its associated health risks. By fostering meaningful social connections and reducing loneliness exposure at the population level, such interventions can create environments that inherently support social well-being.

To be effective, these efforts must be embedded in broader, coordinated, intersectoral policy action—including urban planning, workplace policies, healthcare systems, and community infrastructure. Beyond urban design, policies in housing and transportation offer key entry points to reduce loneliness structurally. Affordable housing initiatives can mitigate residential instability, while accessible, reliable public transportation can facilitate social participation, especially among older adults and low-income populations. This systemic approach prioritizes transforming the social contexts that contribute to loneliness and enables more inclusive, sustainable forms of social connection.

Despite their promise, population-level interventions also face important challenges. Scalability, sustainable funding, and cultural adaptability can limit their effectiveness, and strategies that succeed in one setting may not easily translate to others. Moreover, implementation must proactively address equity concerns to ensure that interventions do not inadvertently reinforce inequalities in access to social resources. Acknowledging these limitations is essential to developing approaches that are evidence-based, context-sensitive, and responsive to the needs of diverse communities. Importantly, while our focus is on population-level approaches, we also acknowledge that individual-level interventions remain essential and complementary for supporting those with severe or entrenched loneliness. For instance, interventions requiring access to safe green spaces or digital literacy may unintentionally exclude low-income or marginalized communities unless designed with explicit equity safeguards.

Ultimately, loneliness is not a personal failing, but a collective challenge that calls for collective solutions. If left unaddressed, loneliness will continue to widen global health inequalities, fragment social cohesion, and deepen structural inequities. However, by reframing loneliness as a public health priority and implementing ambitious population-level, context-sensitive interventions, we have the opportunity not only to reduce its prevalence but also to foster a more equitable society where meaningful social connection is accessible to all. The time for action is now.

## Data Availability

The original contributions presented in the study are included in the article/supplementary material, further inquiries can be directed to the corresponding authors.
